# *Leotia* cf. *lubrica* forms arbutoid mycorrhiza with *Comarostaphylis arbutoides* (Ericaceae)

**DOI:** 10.1007/s00572-014-0590-7

**Published:** 2014-07-18

**Authors:** Katja Kühdorf, B. Münzenberger, D. Begerow, J. Gómez-Laurito, R. F. Hüttl

**Affiliations:** 1 Leibniz Centre for Agricultural Landscape Research (ZALF), Institute of Landscape Biogeochemistry, Eberswalder Straße 84, 15374 Müncheberg, Germany; 2 Ruhr-University of Bochum, AG Geobotany, Universitätsstraße 150, 44780 Bochum, Germany; 3 University of Costa Rica, Escuela de Biología, CP 11501-2060, San José, Costa Rica; 4 Brandenburg University of Technology Cottbus-Senftenberg, Chair of Soil Protection and Recultivation, Box 101344, 03013 Cottbus, Germany; 5 German Research Centre of Geosciences Postsdam (GFZ), Telegrafenberg, 14473 Potsdam, Germany

**Keywords:** Arbutoid mycorrhiza, Anatomy, Morphology, Phylogeny, Costa Rica, Leotiomycetes

## Abstract

Arbutoid mycorrhizal plants are commonly found as understory vegetation in forests worldwide where ectomycorrhiza-forming trees occur. *Comarostaphylis arbutoides* (Ericaceae) is a tropical woody plant and common in tropical Central America. This plant forms arbutoid mycorrhiza, whereas only associations with *Leccinum monticola* as well as *Sebacina* sp. are described so far. We collected arbutoid mycorrhizas of *C. arbutoides* from the Cerro de la Muerte (Cordillera de Talamanca), Costa Rica, where this plant species grows together with *Quercus costaricensis*. We provide here the first evidence of mycorrhizal status for the Ascomycete *Leotia* cf. *lubrica* (Helotiales) that was so far under discussion as saprophyte or mycorrhizal. This fungus formed arbutoid mycorrhiza with *C. arbutoides*. The morphotype was described morphologically and anatomically. *Leotia* cf. *lubrica* was identified using molecular methods, such as sequencing the internal-transcribed spacer (ITS) and the large subunit (LSU) ribosomal DNA regions, as well as phylogenetic analyses. Specific plant primers were used to confirm *C. arbutoides* as the host plant of the leotioid mycorrhiza.

## Introduction

**Fig. 1:**
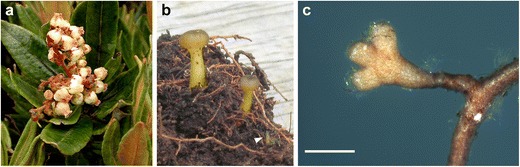
**a** Leafs and flowers of the Ericaceae *Comarostaphylis arbutoides*. Photo by Roy E. Halling. **b** Two fruit bodies and one initial (*arrowhead*) of *Leotia lubrica* found at Estación Biologíca de la Muerte (site I), Costa Rica. Ascomata, 10–40 mm. Photo by Katja Kühdorf. **c** Habit of the mycorrhiza *Leotia* cf. *lubrica*-*Comarostaphylis arbutoides*. Mycorrhiza arbutoid ramified, mantle smooth to moderately hairy, and transparent with foreign brown hyphae; *bar*, 0.5 mm

The tropical woody plant *Comarostaphylis arbutoides* Lindl. (Ericaceae) is common in tropical Central America (c. 2,500–3,430 m height above sea level (a.s.l.)), where it can form extensive thickets. Based on fruit and flower morphology, anatomy, and phytochemistry, this plant is a member of the subfamily Arbutoideae (Hileman et al. 2001; Fig. 1a). The Arbutoideae also include the circumboreal *Arctostaphylos uva-ursi* and species of *Arbutus* that are all known to form arbutoid mycorrhiza (Molina and Trappe 1982a; Münzenberger et al. 1992; Osmundson et al. 2007). The mycorrhizal fungus induces the branching of the lateral roots to form mostly, with some exceptions (Molina and Trappe 1982a), a pinnate-cruciate branching pattern that is typical for this mycorrhizal type (Massicotte et al. 1993). The arbutoid mycorrhiza is characterized by a hyphal mantle, a para-epidermal Hartig net and intracellular hyphae penetrating the living epidermal cells of the host (Münzenberger et al. 1992; Selosse et al. 2007). Suberization of the outer cortical layer leads to the formation of an exodermis and prevents penetration of the fungus into deeper root cell layers (Münzenberger et al. 1992; Massicotte et al. 1993).

*C. arbutoides* is a refuge plant for ectomycorrhizal fungi as it shares these fungi with ectomycorrhizal tropical trees such as *Quercus costaricensis* (Halling and Mueller 2003; Kühdorf et al. 2014). After forest clearcutting of the economically important forest trees, arbutoid mycorrhizal plants host the ectomycorrhizal fungi that contribute to forest recovery later on (Dahlberg 1990; Visser 1995; Molina et al. 1997; Horton et al. 1999; Hagerman et al. 2001). Ectomycorrhizal fungi of Arbutoideae show identical morphology (apart from, e.g., host-depending branching pattern and habit dimensions) and hyphal mantle anatomy as the ectomycorrhiza (ECM) of other hosts such as *Pinus*, *Picea*, *Pseudotsuga*, and *Quercus* (Zak 1976a, b; Molina and Trappe 1982b; Mühlmann and Göbl 2006; Kühdorf et al. 2014). The ericaceous hosts *Arbutus menziesii* and *A. uva-ursi* lack mycorrhizal specificity, which means numerous ectomycorrhizal fungi are able to colonize these ericads (Molina and Trappe 1982a; Acsai and Largent 1983a, b; Massicotte et al. 1994; Richard et al. 2005; Kennedy et al. 2012).

Osmundson et al. (2007) were the first to describe the arbutoid mycorrhiza of *C. arbutoides* and *Leccinum monticola* anatomically and molecularly. To date, only one more morphotype has been described in literature (Kühdorf et al. 2014). However, Halling and Mueller (2004) mention that species of other mycorrhizal genera such as *Tricholoma*, *Cortinarius*, *Russula*, and *Laccaria* are likely associates of *C. arbutoides*.

The ecology of the helotialean fungi is diverse. Members of the Helotiales have been described as plant pathogens, ECM and ericoid mycorrhiza formers, terrestrial and aquatic saprobes, and wood rot fungi (Wang et al. 2006a). The genus *Leotia* (Helotiales) is globally distributed (Wang et al. 2006a, b), whereas the ecological role of the Ascomycete is not clear. Species of *Leotia* and *Microglossum* are found usually on humus-rich soil, sometimes on decaying wood, but rarely on leaf litter (Arora 1986). *Leotia* was already recorded as mycorrhiza-forming fungus by Molina et al. (1992). This status was later speculated to be saprotrophic by Rinaldi et al. (2008) based on insufficient evidence. Both Zeller et al. (2007) and Seitzman et al. (2011) later found the ^13^C signature of the species *L. lubrica* comparable with those of ectomycorrhizal fungi and therefore, conclude that this fungus should be mycorrhizal. Furthermore, *L. lubrica* was reported to form ECM with diverse plant species such as *Quercus rotundifolia*, *Polygonum* sp., and *Nothofagus menziesii* (Branco and Ree 2010; Gao and Yang 2010; Orlovich et al. 2013). However, these records have been proven by DNA sequencing only and were not further described.

The genus *Leotia* contains three species of inoperculate discomycetes with stipitate-capitate ascomata. They are constructed, at least in part, of tissues built-up of hyphae imbedded in a gelatinous matrix (Zhong and Pfister 2004). They are distinguished by the color of their fresh ascomata: species with a yellow stipe and yellow hymenia are assigned to *Leotia lubrica*, those with an entirely green ascomata to *Leotia atrovirens*. Species of *Leotia viscosa* have a yellow stalk and green hymenia (Durand 1908; Mains 1956; Grund and Harrison 1967).

Here, we describe arbutoid mycorrhizal systems morphologically and anatomically according to Agerer (1991), formed by *L*. cf. *lubrica* and *C. arbutoides* from the Cerro de la Muerte (Cordillera de Talamanca), Costa Rica. *L*. cf. *lubrica* was identified using molecular methods such as internal-transcribed spacer (ITS) and large subunit (LSU) sequencing as well as phylogenetic analysis. Plant primers were used to sequence the ITS region of *C. arbutoides* from the same arbutoid mycorrhiza as used for fungal analysis.

## Materials and methods

### Site description and sampling

Sampling was conducted at two forest sites around the Mountain Cerro de la Muerte (3,491 m a.s.l.) in the Cordillera de Talamanca of Costa Rica, 54 km southeast of the capital San José. Both sites are secondary cloud forests and about 1.4 km apart from each other: Estación Biologíca de la Muerte (site I; 3,100 m a.s.l.; 9° 33′ N, 83° 45′ W) and Reserva Forestal Los Santos (site II; 3,300 m a.s.l.; 9° 34′ N, 83° 45′ W). Site I is dominated by *Q. costaricensis* mixed with solitary individuals of *C. arbutoides*. At sampling site II, *C. arbutoides* itself is the dominating species, mixed with a few individuals of *Q. costaricensis*. The understorey species consist of members of the families Araliaceae (*Schefflera* and *Oreopanax*), Cunoniaceae (*Weinmannia*), Ericaceae (*Cavendishia*, *Disterigma,* and *Vaccinium*), Poaceae (*Chusquea*), Primulaceae (*Myrsine*), and Winteraceae (*Drimys*).

Fine root systems of *C. arbutoides* were collected during the rainy seasons in October 2010 and 2011. For this, a soil corer (diameter, 3 cm; length, 40 cm) was used at distances of 50 and 100 cm from the trunk. Within these 2 years, a total of 60 soil cores were taken and analyzed. At the University of Costa Rica, turgid and apparently healthy morphotypes were sorted out using a stereomicroscope. Systems with the same morphological features (e.g., color, hydrophobicity presence, emanating elements, and rhizomorphs) were assigned to one morphotype. For further analyses, the morphotypes were preserved in 2 % glutaraldehyde with 0.1 M sodium cacodylate buffer (Münzenberger et al. 2009) for light microscopy or dried on silica gel for DNA extraction, respectively. Identification of each morphotype is based on their respective sequence type. The genus *Leotia* was proven genetically in six soil cores.

### DNA extraction, PCR, and sequencing

One unramified root tip per morphotype was used for DNA extraction using the DNeasy Plant Mini Kit (Qiagen, Hilden, Germany) following the manufacturer’s recommendations. For phylogenetic analysis at family and species level, the ribosomal nuclear LSU and the ITS region from the ribosomal DNA (rDNA) were amplified. For this purpose, the primer combinations LR0R/LR5 (Moncalvo et al. 2000) and ITS1F/ITS4 (Gardes and Bruns 1993; White et al. 1990) were used. To identify the plant from mycorrhizal roots without co-amplifying fungal DNA, the angiosperm-specific ITS primer pair ITS-5A/ITS-241r was amplified (Osmundson et al. 2007). Direct sequencing of PCR products was performed using the PCR primers as sequencing primers. Sequencing service was facilitated by GATC Biotech AG (Konstanz, Germany).

### Identification and phylogenetic analysis

All fungal sequences obtained for ITS and LSU rDNA were analyzed and edited using Chromas Lite v2.01 software (http://technelysium.com.au). Sequence comparisons were performed in the NCBI database (http://www.ncbi.nlm.nih.gov/) using Megablast, and the database UNITE (Kõljalg et al. 2005; http://unite.ut.ee/) using blastn. To calculate the phylogenetic tree of the ITS region, the 100 most similar sequences for each reference sequence in NCBI database were downloaded and complemented with an additional search in the nucleotide database and sequences of other publications as well. Alignment was performed with the program MAFFT v7 (Katoh et al. 2002) using the FFT-NS-2 alignment algorithm. To estimate phylogenetic relationships, we used maximum likelihood and Bayesian approaches. Maximum likelihood analyses was performed using RAxML (v7.3.2; Stamatakis 2006; Stamatakis et al. 2008) in a parallelized version supplied by Bioportal (http://bioportal.uio.no/) with eight parallel processors and trees inferred from 10,000 rapid bootstrap analyses as starting trees in a heuristic search for the tree with the highest likelihood. GTRCAT was used in the heuristic search and the final evaluation of the best tree found was based on the GTR + Gamma model. The Bayesian analyses were performed using MrBayes v3.2.1 (Ronquist et al. 2012) on an iMac (2.9 GHz Quad-Core Intel Core i5). The GTR + Gamma model was in effect, and four chains in two parallel runs were performed for 2,000,000 generations. The first 50,000 trees were discarded before calculating the posterior probabilities.

### Microscopy

The morphological and anatomical description of the mycorrhizas was carried out according to Agerer (1987–2012, 1991), and using the online key of DEEMY (Agerer and Rambold 2004–2014). The iodine reaction specially adapted to Ascomycetes was done after Baral (1987, 2009). Anatomical studies are based on 15 mycorrhizal systems. Drawings were performed by using an interference contrast microscope (BX50F-3, Olympus Corporation, Tokyo, Japan) connected with a drawing tube. All drawings were made at a thousand-fold magnification.

For semi-thin sections, the mycorrhizas were fixed with 2 % glutaraldehyde in 0.1 M sodium cacodylate buffer (pH 7.2) at room temperature until further processing. Thereafter, six washes in 0.1 M sodium cacodylate buffer were performed. Samples were postfixed in 1 % osmium tetroxide in the same buffer for 1 h under light exclusion at room temperature. After six washes in double-distilled water, samples were dehydrated by immersion for 15 min each in 25, 50, 70, and 95 % acetone and three times for 1 h in 100 % acetone (Münzenberger et al. 2009). The mycorrhizal tips were embedded in Spurr’s plastic (Spurr 1969) and sectioned with a diamond knife on an Ultracut Reichert Ultramicrotome (W. Reichert-LABTEC, Wolfratshausen, Germany). The sections (0.5 μm thick) were stained with crystal violet and investigated by use of a light microscope (Zeiss Axioskop 50, Oberkochen, Germany).

### Fruit bodies of *L. lubrica*

During 2 years of sampling, fruit bodies of *L. lubrica* were found only once and were coincidentally documented. For that reason, no further genetic investigations were carried out to clearly identify the fungus. As the three species of the genus *Leotia* are easy to distinguish morphologically, the ascomata (Fig. 1b) were preliminary identified as *L. lubrica*, based on the yellow color of both the stipe and the hymenium.

## Results

### Morpho-anatomical description of the mycorrhiza *Leotia* cf. *lubrica*-*C. arbutoides*

#### Morphological characters

(Fig. 1c) *Mycorrhizal systems* arbutoid, with 0–1 (4) orders of ramification, solitary or in small numbers, up to 2.8 mm long, main axis at 0.3 mm diameter; mantle surface hydrophilic and smooth to moderately hairy, of contact exploration type or short-distance exploration type. *Unramified ends* straight to slightly bent, cylindric, not inflated, 0.1–0.7 (1.4) μm long, 0.2–0.3 (0.1) μm diameter, mantle consistently transparent and yellowish to light orange, older parts dark orange to ochre. *Surface of unramified ends* smooth to occasionally hairy, cortical cells (correspond to epidermal cells) visible. *Cystidia* not distinct under stereomicroscope magnification. *Rhizomorphs* not found. *Sclerotia* not observed.

#### Anatomical characters of the mantle in plan views

**Fig. 2:**
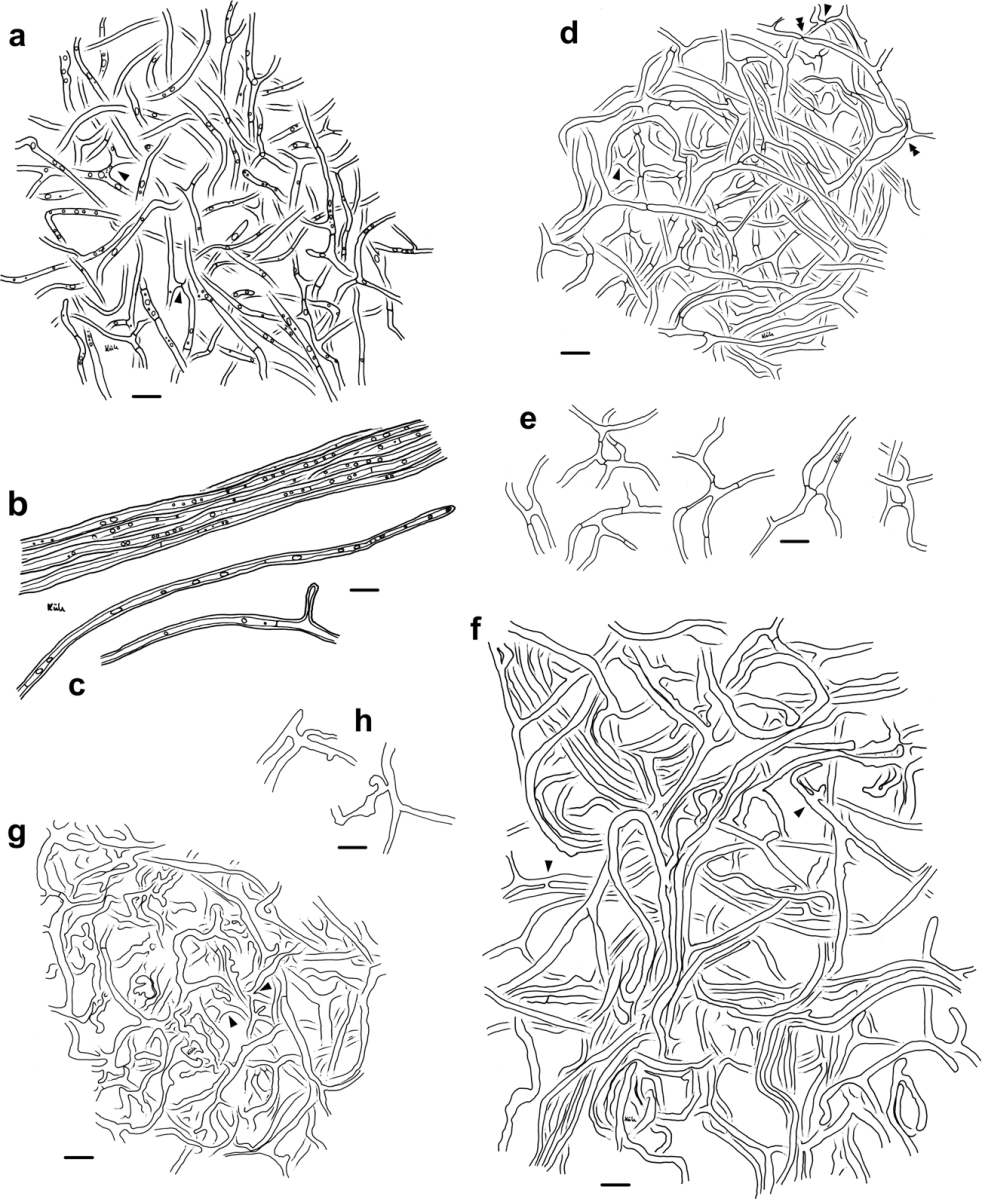
Arbutoid mycorrhiza of *Leotia* cf. *lubrica*-*Comarostaphylis arbutoides*. **a**–**f** Plan view of different mantle layers, emanating hyphae and anastomoses; *bars*, 10 μm: **a** outer mantle layer with ramified hyphae, irregularly arranged; hyphae with numerous oily droplets; anastomoses with closed long bridge (*arrowheads*); **b** bundle of emanating hyphae, hyphae with oily droplets; **c** simple and branched emanating hyphae; **d** middle mantle layer with repeatedly branched hyphae, densely arranged; anastomoses with short or long bridge, bridge closed or open (*single arrowheads*); merged hyphal tips, some with remnants of a partly solved septum (*double arrowheads*); **e** different anastomoses of the middle mantle layer; **f** inner mantle layer with ring-like arrangement of hyphal bundles, some hyphae irregularly inflated; anastomoses (*arrowheads*); **g** inner mantle layer close to the very tip with many irregularly inflated hyphae, many hyphae with hyphal tips; different anastomoses (*arrowheads*); **h** anastomoses of both the inner mantle layer and the inner mantle layer close to very tip, anastomosis close to the hyphal tip

**Fig. 3:**
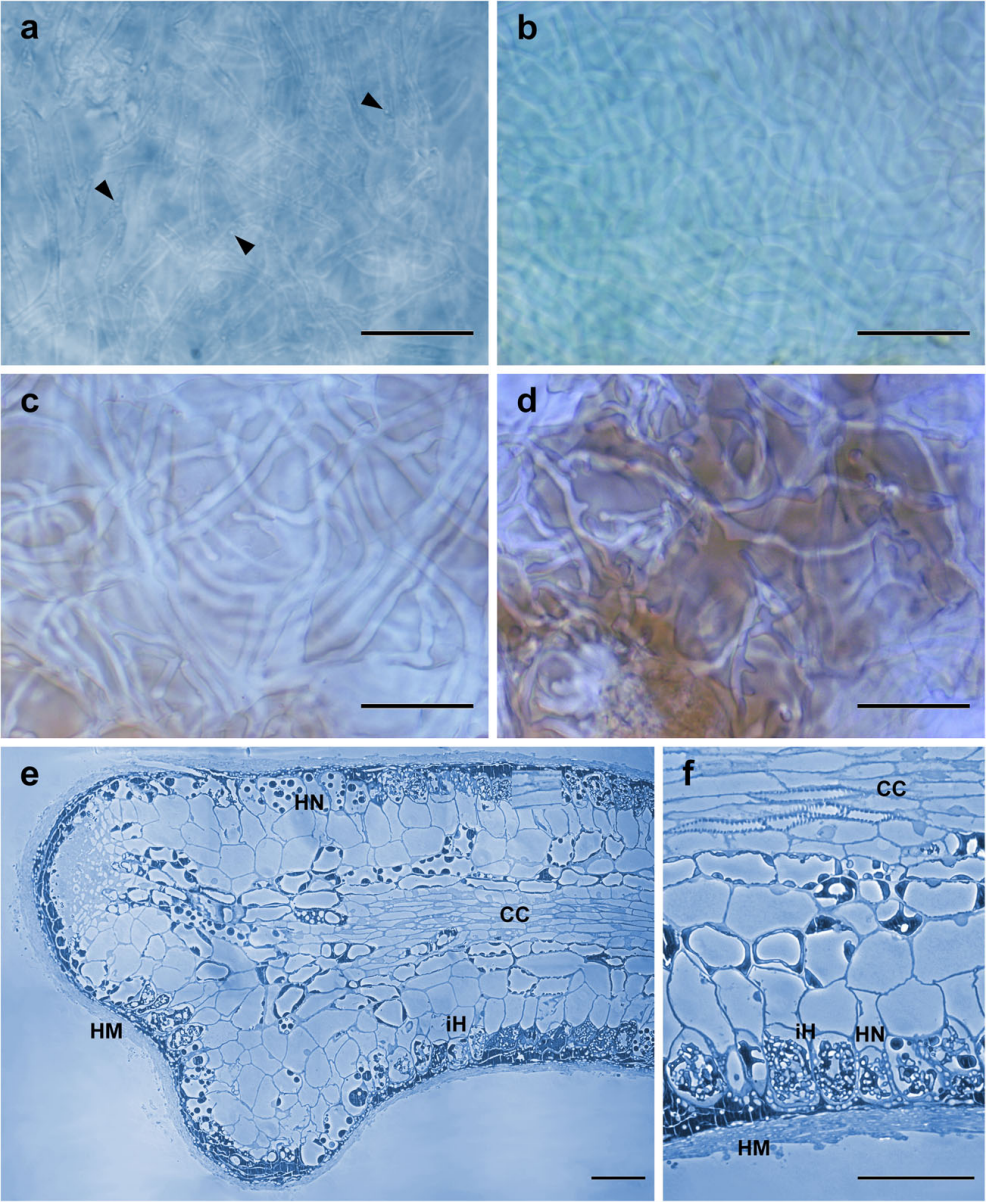
**a**–**d** Interference contrast of the three mantle layers of leotioid mycorrhiza; *bars*, 20 μm: **a** outer mantle layer, hyphae containing numerous oily droplets (*arrowheads*). **b** Middle mantle layer. **c** Inner mantle layer. **d** Inner mantle layer close to very tip with brownish matrix (remnants of root cap cells). **e**–**f** Semi-thin sections of the arbutoid mycorrhiza *Leotia* cf. *lubrica*-*Comarostaphylis arbutoides*; *bars*, 50 μm: **e** hyphal mantle (*HM*), Hartig net (*HN*), intracellular hyphae (*iH*), and central cylinder (*CC*). **f** Anatomical features in detail

 (Figs. 2 and 3a–d) Mantle plectenchymatous throughout, all hyphae clampless and smooth; laticifers are lacking. *Outer mantle layers* (Figs. 2a and 3a) hyphae arranged net-like; hyphae frequently branched, often with merged hyphal tips, matrix lacking (mantle type E; Agerer 1991); hyphae septate, even and straight, not constricted at septa; hyphae with closed anastomoses, anastomosal bridge long, mostly thinner than hyphae; hyphae with numerous oily droplets, droplets light yellow to light orange; cytoplasm colorless; hyphae 10–90 μm long, 0.7–3.1 μm diameter; cell walls 0.2–0.4 μm thick; septa as thick as cell walls and often difficult to discern due to frequent droplets. *Middle mantle layers* (Figs. 2d, e and 3b) contain a nongelatinous matrix, hyphae irregularly interwoven, repeatedly branched and septate, colorless, no discernible pattern; frequently merged hyphal tips, hyphae at distal end simple; hyphae 15–50 (90) μm long, 0.9–2.4 μm diameter; cell walls 0.2 μm thick; septa as thick as cell walls; anastomoses frequently and very variable in shape; anastomoses open or closed by a simple septum; anastomosal bridge long, short, or almost lacking; bridge thinner or thicker than hyphae or as thick as hyphae; cell walls of anastomoses as thick as remaining wall; anastomoses close to hyphal tips not found. *Inner mantle layers* (Figs. 2f, h and 3c) ring-like arrangement of hyphal bundles; hyphae even or irregularly inflated, at distal end simple or slightly inflated; hyphae rarely septate, colorless; hyphae (7) 20–130 μm long, 1.5–3.9 μm diameter; cell walls 0.2–0.3 μm thick, septa as thick as cell walls; anastomoses frequently, anastomoses open, with short bridge or bridge almost lacking, bridge thinner or thicker than hyphae, or as thick as hyphae; cell walls of anastomoses as thick as remaining walls; anastomoses close to hyphal tips also present. *Very tip* (Figs. 2g, h and 3d) inner mantle layers similar to remaining part but without hyphal bundles; hyphae often irregularly inflated, many hyphae with hyphal tips; outer and middle mantle layers organized as older parts of the mantle.

#### Anatomical characters of emanating elements

(Fig. 2b, c) *Rhizomorphs* lacking. *Emanating hyphae* rare to frequently, not specifically distributed; hyphae even, not striking, sometimes one-side branched, sometimes with open anastomoses; hyphal tips sometimes merged with hyphae; some hyphae in bundles; hyphae at distal end simple; hyphae straight and even, up to 500 μm long, maybe longer since often observed without hyphal tip, 2.3–4.2 μm diameter; cell walls 0.5–1.2 μm thick, not constricted at septa, septa 0.2–0.3 (0.6) μm thick, septa often difficult to discern due to frequent droplets; cell wall light yellow to light orange, cytoplasm light dirty blue, oily droplets same color as cell wall; lacking are clamps, elbow-like protrusions, and intrahyphal hyphae. *Cystidia* not found.

#### Anatomical characters of longitudinal section

(Fig. 3e, f) Mantle plectenchymatous, 5–17 μm thick. Mantle of very tip plectenchymatous, 8–20 μm thick. Epidermal layer with intracellular hyphae, epidermal cells radially oval to eliptic; Hartig net around epidermal cells para-epidermal in one row; hyphal cells roundish to cylindrical. Tannin cells lacking.

#### Color reactions with different reagents (mantle preparations and emanating hyphae)

Acetic acid: no reaction; congo red: no reaction; cotton blue: hyphae of outer mantle layer with blue cytoplasm and blue (sometimes reddish) oily droplets, emanating hyphae with violet (sometimes reddish) cell walls, blue cytoplasm, and red to red brown oily droplets; ethanol 70 %: no reaction; Fe(II)SO_4_: no reaction; guaiac: no reaction; KOH 10 %: no reaction; lactic acid: no reaction; Lugol’s solution: no reaction; Melzer’s reagent: no reaction; NH_4_OH: no reaction; sulpho-vanillin: no reaction; H_2_SO_4_ concentration: no reaction; toluidine blue: hyphae of outer mantle layer with patchy pale blue to blue cytoplasm and red oily droplets, emanating hyphae with violet cell walls, patchy pale blue to blue cytoplasm, and red oily droplets.

#### Reference specimen

Costa Rica, province of San José, canton of Pérez Zeledón, at mountain Cerro de la Muerte, Reserva Forestal Los Santos (3,300 m a.s.l.; precipitation c. 2,812 mm/year; inceptisol (USDA)), in a secondary cloud forest with *Q. costaricensis*, soil core exc., myc. isol. Katja Kühdorf; KKM 337 and KKM 348, 18 October 2011; mycorrhiza deposited by B. Münzenberger (ZALF Müncheberg, Germany). *Further material studied* same location, soil core exc., myc. isol. Katja Kühdorf; KKM 334 and KKM 347, 18 October 2011; mycorrhiza deposited by B. Münzenberger (ZALF Müncheberg, Germany).

### Phylogenetic analyses

A total of 399 root tips were analyzed genetically, of which ten were identified as *Leotia* cf. *lubrica*. All leotioid sequences were deposited in NCBI GenBank under the accession numbers KF836622-KF836631 (LSU) and KF836612-KF836621 (ITS), respectively. In all samples, *C. arbutoides* (KF419121) was proven as host tree.

The sequenced LSU rDNA region of the ten arbutoid mycorrhizas of *C. arbutoides* resulted in sequences with a length of 890–913 bp (KF836622–KF836631), in which the overlapping area was different in five positions within these samples. Sequence comparison with BLASTn in NCBI database resulted in matches mainly belonging to the order Helotiales, where the Leotiaceae *L. lubrica* (AY789359) showed the highest similarity values (Table 1). In UNITE, best values were reached with the Helotiaceae *Unguiculariopsis thallophila* (UDB016232), which also belongs to the Helotiales. In the sequenced 563–616 bp long ITS region (KF836612-KF836621) of the arbutoid mycorrhizas of *C. arbutoides*, 30 positions were different in the overlapping area. Sequences obtained from NCBI and UNITE comparison with ITS sequences belong to members of the genus *Leotia* (Table 1), whereby UNITE provides lower similarity values.

**Table 1:** Comparison of ITS and LSU sequences with NCBI and UNITE database obtained from ten mycobionts of *Comarostaphylis arbutoides*

Samples with accession numbers (ITS; LSU)	NCBI (ITS)				UNITE (ITS)				NCBI (LSU)				UNITE (LSU)			
	Closest match^a^	Highest maximum score	*E* value/query coverage (%)	Identity (%)	Closest match^a^	Highest bit-score	*E* value/query coverage (%)	Identity (%)	Closest match^a^	Highest maximum score	*E* value/query coverage (%)	Identity (%)	Closest match^a^	Highest bit-score	*E* value/query coverage (%)	Identity (%)
KKM 145 (KF836612; KF836622)	*Leotia lubrica* (EU819412)	1,072	0.0/99	99	*Leotia* sp. (UDB013464)	504	e − 143/56	94	*Leotia lubrica* (AY789359)	1,650	0.0/100	99	*Unguiculariopsis thallophila* (UDB016232)	985	0.0/–^a^	–^a^
KKM 147 (KF836613; KF836623)	*Leotia viscosa* (AY144536)	942	0.0/100	97	*Leotia* sp. (UDB013464)	478	e − 135/60	94	*Leotia lubrica* (AY789359)	1,644	0.0/100	99	*Unguiculariopsis thallophila* (UDB016232)	979	0.0/–^a^	–^a^
KKM 152 (KF836614; KF836624)	*Leotia lubrica* (AY144544)	1,064	0.0/99	99	*Leotia* sp. (UDB013464)	496	e − 140/56	94	*Leotia lubrica* (AY789359)	1,644	0.0/100	99	*Unguiculariopsis thallophila* (UDB016232)	977	0.0/–^a^	–^a^
	*Leotia lubrica* (AY144551)	1,064	0.0/99	99												
KKM 317 (KF836615; KF836625)	*Leotia lubrica* (AY144547)	1,083	0.0/97	99	*Leotia* sp. (UDB013464)	533	e − 152/55	95	*Leotia lubrica* (AY789359)	1,631	0.0/99	99	*Unguiculariopsis thallophila* (UDB016232)	971	0.0/–^a^	–^a^
	*Leotia lubrica* (AY144548)	1,083	0.0/97	99												
KKM 334 (KF836616; KF836626)	*Leotia lubrica* (EU819412)	1,092	0.0/99	99	*Leotia* sp. (UDB013464)	496	e − 140/55	94	*Leotia lubrica* (AY789359)	1,639	0.0/99	99	*Unguiculariopsis thallophila* (UDB016232)	971	0.0/–^a^	–^a^
KKM 337 (KF836617; KF836627)	*Leotia lubrica* (EU819412)	1,092	0.0/99	99	*Leotia* sp. (UDB013464)	496	e − 140/55	94	*Leotia lubrica* (AY789359)	1,629	0.0/100	99	*Unguiculariopsis thallophila* (UDB016232)	961	0.0/–^a^	–^a^
KKM 347 (KF836618; KF836628)	*Leotia lubrica* (EU819412)	1,022	0.0/99	99	*Leotia* sp. (UDB013464)	496	e − 140/58	94	*Leotia lubrica* (AY789359)	1,604	0.0/100	99	*Unguiculariopsis thallophila* (UDB016232)	938	0.0/–^a^	–^a^
KKM 348 (KF836619; KF836629)	*Leotia lubrica* (EU819412)	1,092	0.0/99	99	*Leotia* sp. (UDB013464)	496	e − 140/55	94	*Leotia lubrica* (AY789359)	1,602	0.0/100	99	*Unguiculariopsis thallophila* (UDB016232)	938	0.0/–^a^	–^a^
KKM 349 (KF836620; KF836630)	*Leotia lubrica* (EU819412)	1,024	0.0/99	99	*Leotia* sp. (UDB013464)	496	e − 140/58	94	*Leotia lubrica* (AY789359)	1,602	0.0/100	99	*Unguiculariopsis thallophila* (UDB016232)	938	0.0/–^a^	–^a^
KKM 427 (KF836621; KF836631)	*Leotia lubrica* (EU819412)	1,092	0.0/99	99	*Leotia* sp. (UDB013464)	496	e − 140/55	94	*Leotia lubrica* (AY789359)	1,637	0.0/100	99	*Unguiculariopsis thallophila* (UDB016232)	969	0.0/–^a^	–^a^

Closest match was chosen according to the highest maximum score or bit-score

^a^Information locked by the reference author

**Fig. 4:**
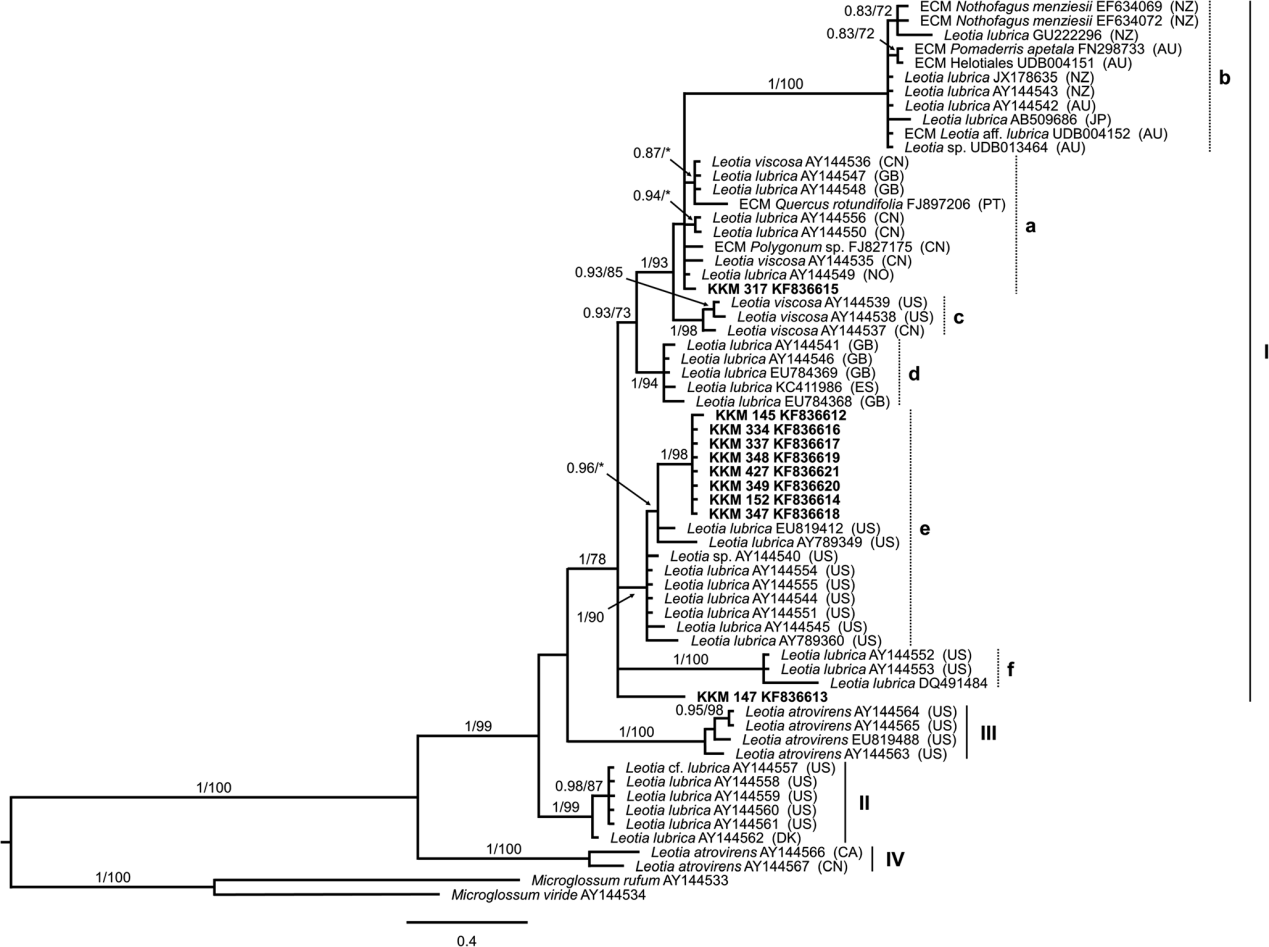
Phylogenetic relationship of ten leotioid mycobionts of *Comarostaphylis arbutoides* within the genus *Leotia*. Phylogram was obtained from Bayesian analysis based on ITS sequences. Branch support values were calculated as posterior probability from 2,000,000 generations of Bayesian analysis (*first number*) and as bootstrap support from RAxML analysis (*second number*). Values below 70 % are indicated with *asterisks* or omitted. The phylogram was rooted with *Microglossum rufum* and *M. viride*. Sequences were obtained from NCBI and UNITE database complemented by the name of corresponding host plant, if available. Investigated arbutoid mycobionts of *C. arbutoides* from Costa Rica are marked in *bold*. Mycorrhizal type: ectomycorrhiza (*ECM*). Country codes, if applicable: Australia (*AU*), Canada (*CA*), China (*CN*), Denmark (*DK*), Spain (*ES*), UK (*GB*), Japan (*JP*), Norway (*NO*), New Zealand (*NZ*), Portugal (*PT*), and USA (*US*). Indicated groups (*I*–*IV*) and subgroups (*a*–*f*) after Zhong and Pfister (2004)

The Bayesian and RAxML phylogenies, generated by ITS sequences are concordant. Both trees show the same grouping structure, supported by high posterior probabilities (PP) in the Bayesian analysis and by typically lower bootstraps (BS) in the RAxML analysis (Fig. 4). The phylogenetic analysis in Fig. 4 reveals that the three species of the genus *Leotia* are paraphyletic and split in several groups. Members of the species *L. atrovirens* can be found in two groups (III and IV), whereby both are highly supported (PP 1/BS 100, each). Samples of *L. lubrica* are also divided and can be found exclusively in group II (PP 1/BS 99) on the one hand, and as a large complex together with *L. viscosa* samples in group I (PP 1/BS 78) on the other. In this *L. lubrica*/*viscosa* complex, again several subgroups (a–f) are formed, containing exclusively *L. lubrica* (b, d, e, f; PP 1/BS 90-100) and *L. viscosa* (c; PP 1/BS 98) species, respectively, as well as a mixture of *L. lubrica* and *L. viscosa* samples (a).

All investigated leotioid arbutoid mycorrhizas of *C. arbutoides* can be found in group I, whereby eight group together (PP 1/BS 98) and nest within samples of *L. lubrica* (subgroup e; PP 1/BS 90). Therefore, these samples were identified as *L*. cf. *lubrica* species. The other two mycorrhizas do not group to a specific *Leotia* species. KKM 317 (KF836615) can be found among various *L. lubrica* and *L. viscosa* samples within subgroup a, which is not well supported, whereas the sample KKM 147 (KF836613) does not cluster in one of the above mentioned subgroups.

## Discussion

The classification of Ascomycota was traditionally based on the morphology of their fruit bodies, whereas, molecular studies showed that such morphologically defined groups can be phylogenetically misleading (Wang et al. 2006a, b). Currently, the five orders Cyttariales, Erysiphales, Helotiales, Rhytismatales, and Thelebolales are placed in the ascomycetous class Leotiomycetes (Hibbett et al. 2007). The genus *Leotia* Pers. belongs to the Leotiaceae (Helotiales) whose final composition of genera is not fully resolved. Currently, Lumbsch and Huhndorf (2009) place the genera *Geocoryne*, *Gelatinipulvinella*, *Leotia*, *Microglossum*, *Neobulgaria*, and *Pezoloma* into this family. Sufficient molecular information of currently included genera is rarely or not at all available in NCBI database. Therefore, further reinterpretations within the Leotiaceae family are expected. Nevertheless, the obtained LSU rDNA sequences of the ten arbutoid mycorrhizas of *C. arbutoides* (KF836622–KF836631) identify them as members of the genus *Leotia* (Table 1).

Thus far, a detailed phylogenetic investigation within the genus *Leotia* has been carried out only by Zhong and Pfister (2004). They combined morphological information with phylogenetic analyses and found four groups within the genus *Leotia*. Group I comprises all *L. viscosa* and some *L. lubrica* samples, which are characterized by a yellow stipe in fresh and dry condition, respectively. Another group (II) exclusively formed by *L. lubrica* samples, however, showed a green stipe when dry. The species *L. atrovirens* is divided into two groups (III and IV) and differ from each other by the presence or absence of gel in their stipes.

Genetically, eight of ten arbutoid mycorrhizas of *C. arbutoides* collected in Costa Rica were identified as *L*. cf. *lubrica*. The other two leotioid mycorrhizas samples KKM 147 (KF836613) and KKM 317 (KF836615) still remain unidentified, whereby the first one represents a further genotype within the *L. lubrica*/*viscosa* complex, because it does not cluster in a specific subgroup within group I. However, those two leotioid mycorrhizas are also assumed to be *L. lubrica* species, since they were found as mycorrhizal partner of *C. arbutoides*. This suggests a high genetic variability of *L. lubrica* species assigned to group I.

The stipes of the *L. lubrica* fruit bodies found at site I, were not further investigated regarding a possible color change in dried condition or genetically. As pointed out by Zhong and Pfister (2004), it is difficult to distinguish between *L. lubrica* species of group I and II, as both have a yellow stipe in fresh conditions. Thus, it is not possible to clearly assign the found fruit bodies to one of both groups. However, thus far mycorrhizal associations are only known from group I *L. lubrica* samples, not from *L. lubrica* species belonging to group II. Among *C. arbutoides*, the species *Q. rotundifolia*, *Polygonum* sp., and *N. menziesii* are reported as host plants of leotioid ECMs (Branco and Ree 2010; Gao and Yang 2010; Orlovich et al. 2013). Orlovich et al. (2013) additionally indicate that *Leotia* perhaps interact in some way with other ectomycorrhizal fungi, since the fungus was found with either *Russula*, *Clavulina*, or *Laccaria* at the same root tip. Tedersoo et al. (2009) identified *L. lubrica* from ectomycorrhizal roots formed by Basidiomycota and the Rhamnaceae *Pomaderris apetala* (FN298733) and suggest a secondary colonization of ECMs by this fungal species (Tedersoo et al. 2010). However, such secondary mycorrhizal association for the described leotioid arbutoid mycorrhizas of *C. arbutoides* is not assumed. Color of leotioid mycorrhiza is similar to the fruit bodies of *L. lubrica* and all investigated samples show the same morpho- and anatomotype. Additionally, semi-thin sections reveal a mantle, Hartig net, as well as intracellular hyphae, typical for mycorrhizas of the Arbutoideae (Fig. 3e, f). However, only samples of *L*. cf. *lubrica* clustering in subgroup e were investigated, so no conclusion can be made if there are morphological or anatomical differences regarding the leotioid mycorrhizas KKM 147 (KF836613) and KKM 317 (KF836615).

The *L*. cf. *lubrica* arbutoid mycorrhiza is morphologically characterized by a hydrophilic, consistently transparent and yellowish colored mantle. According to Agerer and Rambold (2004–2014), these features are in common with the ECM of the Basidiomycete *Entoloma nitidum* (Montecchio et al. 2006). Given their smooth to moderately hairy surface, the leotioid arbutoid mycorrhiza is assigned to the contact exploration type or short distance exploration type (Agerer 2001). By contrast, the mantle of the *E. nitidum* ECM features abundant rhizomorphs and is therefore assigned to the medium distance exploration type (Montecchio et al. 2006). Anatomically, all mantle layers lack a matrix and offer hyphae with clamps, which make it also different from the leotioid mycorrhiza.

Anatomically, the *L*. cf. *lubrica* arbutoid mycorrhiza is characterized by a continuous plectenchymatous mantle, wherein the middle mantle layer is embedded in a nongelatinous matrix. Anastomoses can be found in all layers, showing various types. Emanating hyphae also show anastomoses, which confirm them as such structures and not as cystidia (Agerer 1999). The yellowish droplets are another characteristic feature, only found in emanating hyphae and in hyphae of the outer mantle layer. The ECM of the hypogeous Ascomycete *Gautieria inapire* (Palfner and Horak 2001) also show a plectenchymatous mantle in all layers with clampless hyphae and oily droplets, which do not stain in sulpho-vanillin (Agerer and Rambold 2004–2014). However, it differs from *L*. cf. *lubrica* arbutoid mycorrhiza in having cystidia, emanating hyphae, and rhizomorphs. A matrix is also present, but shows, by contrast, a gelatinous condition and can be found only in the inner mantle layer.

Color reactions of the leotioid arbutoid mycorrhiza are observed with cotton blue and toluidine blue, which are restricted to the hyphae of the outer mantle layer, emanating hyphae, and the droplets within. Both chemicals cause a metachromatic reaction due to the color change of droplets to red. Additionally, cell walls of the emanating hyphae turn violet in cotton blue and are thus cyanophil. In contrast, other applied chemicals cause no reaction, for instance, dissolution of the droplets.

Evidence of amyloidity or dextrinoidity in fungal structures is an important characteristic feature in taxonomy (Baral 1987; Agerer and Rambold 2004–2014). These blue (amyloid) or red to red-brown (dextrinoid) iodine-based reactions are induced by Melzer’s reagent as well as Lugol’s solution. According to Baral (1987) there exists a special case of amyloidity, called hemiamyloidity. Here, Lugol’s solution provokes a red reaction, whereas Melzer’s reagent yields no reaction at all due to chloral hydrate contained within. However, a pretreatment with KOH causes a blue reaction in both, Lugol’s and Melzer’s. This hemiamyloid color reaction is so far only known in Ascomycetes, including many Helotiales (Baral 2009). Among mycologists, Melzer’s reagent is prefered over Lugol’s solution. This makes it more difficult to observe clearly a hemiamyloid reaction as Melzer’s reagent solely used may falsely indicate inamyloidity (Baral 2009). For that reason, a possible hemiamyloid reaction of Ascomycete mycorrhizas could be overlooked. Regardless which treatment was applied, the arbutoid mycorrhiza of *L*. cf. *lubrica* reacts inamyloid. Zhong and Pfister (2004) confirm this reaction also for the cap of the ascoma for all three species of the genus *Leotia*.

The present study is the first morpho-anatomical proof that the species *L*. cf. *lubrica* is actually mycorrhizal. Besides *C. arbutoides* (Ericaceae) as mycorrhizal partner, an association likewise with *Q. costaricensis* has not been found as it was shown for the Basidiomycete *Sebacina* sp. (Kühdorf et al. 2014). However, an ectomycorrhizal association with *Q. costaricensis* is likewise assumed, since Branco and Ree (2010) reported a leotioid ECM with *Q. rotundifolia*. Nevertheless, it is interesting that mycorrhizas of *L. lubrica* were overlooked so far, although the fungus occurs worldwide. One possibility could be a low competitive ability, e.g., due to slow colonization of root tips (Kennedy 2010). In addition, a dual lifestyle (mycorrhizal as well as saprotrophic) may be also possible, as is known for the ectomycorrhizal Basidiomycete *Laccaria bicolor* (Vincent et al. 2012). However, these assumptions need further investigation. Further sampling could reveal if group II *L. lubrica* species also have the potential to be mycorrhizal or if this ability is limited to members of group I.
